# The Role of the Lateral Habenula in Inhibitory Learning from Reward Omission

**DOI:** 10.1523/ENEURO.0016-21.2021

**Published:** 2021-06-16

**Authors:** Rodrigo Sosa, Jesús Mata-Luévanos, Mario Buenrostro-Jáuregui

**Affiliations:** ^1^Universidad Panamericana, Escuela de Pedagogía, 49 Álvaro del Portillo, Ciudad Granja, Zapopan, 45010, Mexico; ^2^Universidad Iberoamericana, Laboratorio de Neurociencias, 880 Prolongación Paseo de la Reforma, Lomas de Santa Fé, Mexico City, 01219, Mexico

**Keywords:** dopamine signaling, conditioned inhibition, inhibitory control, mesolimbic pathway, mesocortical pathway, negative reward prediction error

## Abstract

The lateral habenula (LHb) is a phylogenetically primitive brain structure that plays a key role in learning to inhibit distinct responses to specific stimuli. This structure is activated by primary aversive stimuli, cues predicting an imminent aversive event, unexpected reward omissions, and cues associated with the omission of an expected reward. The most widely described physiological effect of LHb activation is acutely suppressing midbrain dopaminergic signaling. However, recent studies have identified multiple means by which the LHb promotes this effect as well as other mechanisms of action. These findings reveal the complex nature of LHb circuitry. The present paper reviews the role of this structure in learning from reward omission. We approach this topic from the perspective of computational models of behavioral change that account for inhibitory learning to frame key findings. Such findings are drawn from recent behavioral neuroscience studies that use novel brain imaging, stimulation, ablation, and reversible inactivation techniques. Further research and conceptual work are needed to clarify the nature of the mechanisms related to updating motivated behavior in which the LHb is involved. As yet, there is little understanding of whether such mechanisms are parallel or complementary to the well-known modulatory function of the more recently evolved prefrontal cortex.

## Significance Statement

The lateral habenula (LHb) is a brain structure that has received a great deal of attention and has been a hot topic in the past decades. Consequently, this research field has been extensively reviewed. We review in detail some key recent findings that are pivotal in framing the role of the LHb in well-described associative learning phenomenon, conditioned inhibition. This specific topic has not been considered deeply enough in previous review articles. We also outline the possible mechanisms by which the LHb updates behavior by means of two identified pathway categories (inhibitory and excitatory). This provides a comprehensive account potentially embracing more issues than previously thought, refines our understanding of multiple reward-related mechanisms, and raises novel research questions.

## Introduction

The lateral habenula (LHb) is a phylogenetically preserved brain structure located in the dorsomedial surface of the thalamus that participates in learning from aversive (i.e., undesired) experiences. These include primary aversive stimuli, reward omission, and cues associated with either aversive stimuli or reward omission (Baker et al., 2016). However, latest research suggests that the LHb is not as crucial for learning from primary aversive experiences (see Li et al., 2019) as it is for learning from reward omission.

**Figure 1.**
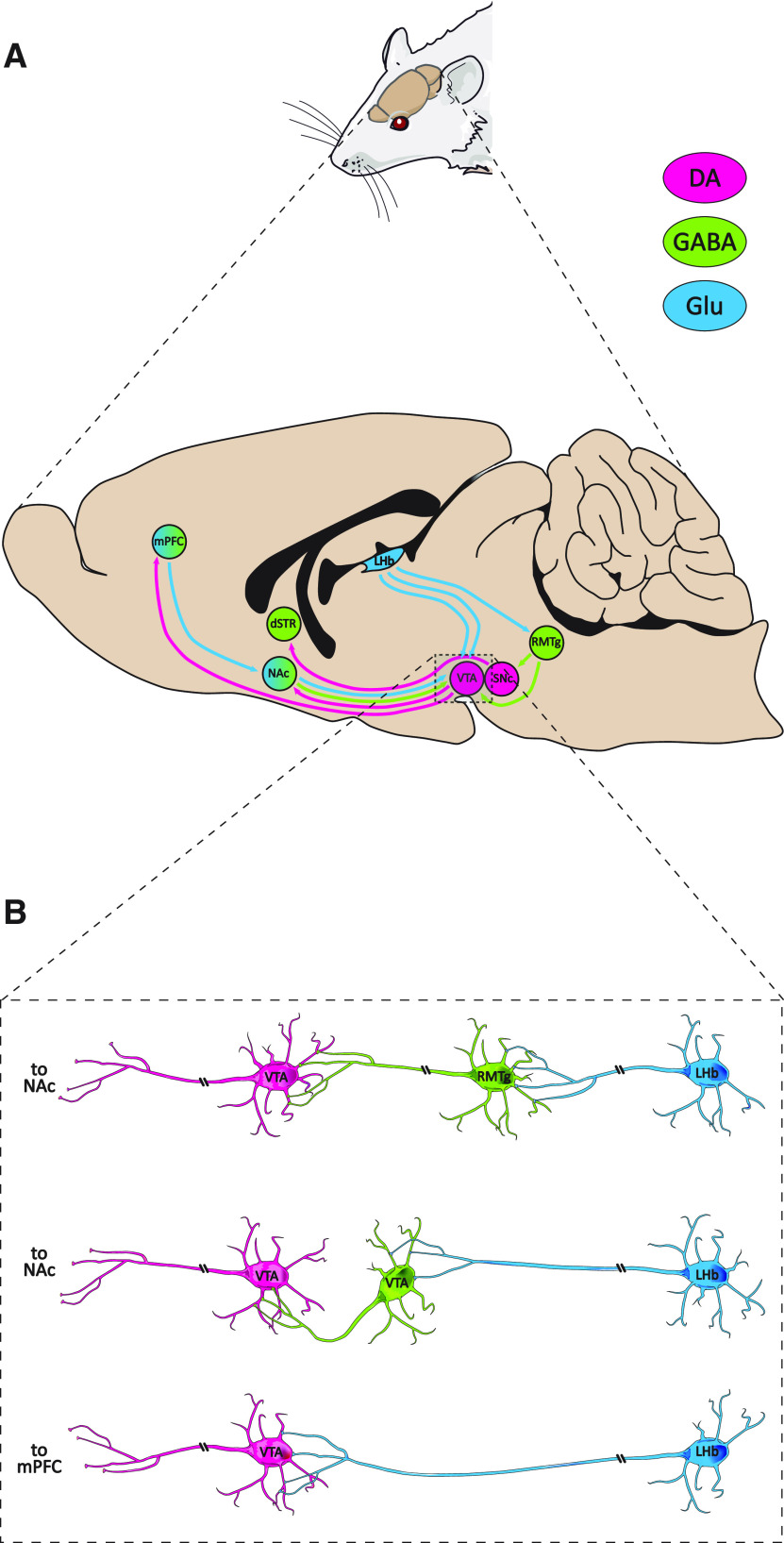
Projections of the midbrain dopamine regions and their neurochemical interaction with the LHb. ***A***, Sagittal view of the rat brain, depicting efferent pathways of midbrain dopamine regions and their input from the LHb. ***B***, Synaptic relationships between neurons in the VTA and inputs (direct and through the RMTg) from the LHb. DA, dopamine (orange); GABA, green; Glu, glutamate (blue); dSTR, dorsal striatum. The image of the rat was adapted from https://smart.servier.com/smart_image/rat, under the terms of the CC-BY 3.0 Unported license (https://creativecommons.org/licenses/by/3.0/). Rat brain adapted from Juarez et al. (2013).

The LHb has been characterized as a part of a “brake” mechanism to suppress firing in midbrain dopaminergic neurons (Barrot et al., 2012; Vento and Jhou, 2020). Such effect is mainly achieved by exciting GABAergic neurons in the rostromedial tegmental nucleus (RMTg) reaching the ventral tegmental area (VTA) and the substantia nigra pars compacta (SNc; Jhou et al., 2009). However, direct glutamatergic excitation of GABAergic interneurons that synapse with dopamine neurons in the VTA also takes place (Omelchenko and Sesack, 2009). In both cases, the net effect is to suppress dopamine release in the nucleus accumbens (NAc; by VTA endings) and the dorsal striatum (by SNc endings). This would disrupt reward-related motor activity, and reward-related plastic changes associated with midbrain dopamine release (Tsutsui-Kimura et al., 2020). A third, recently discovered, pathway (Lammel et al., 2012) involves LHb glutamatergic projections reaching dopamine neurons in the VTA that, in turn, target the medial prefrontal cortex (mPFC; see Fig. 1). mPFC activity has been associated with aversive learning (Huang et al., 2019) and the capacity to behave congruently with hierarchically arranged stimuli sets (Roughley and Killcross, 2019).

Numerous reviews about the physiology of the LHb have already been published; most of them cover the participation of the LHb in primary aversive learning, stressing on the its putative implications for psychiatric conditions (Hu et al., 2020). Other settings that involve the LHb in non-primary aversive situations, such as tasks that require behavior flexibility, have also been reviewed (Baker et al., 2016). In this review, we discuss the experimental findings on a third ground, the role of the LHb in reward-omission learning, which is known to imply negative reward prediction errors. Particularly, we explore how such findings could be framed in terms of some models of behavioral change. This might be informative for a research agenda aiming to unveil the functional basis of adaptive mechanisms in which the LHb participates. In addition, this may aid in understanding maladaptive behaviors that arise from the disruption of brain regions in the extended network in which the LHb is embedded. We conceive learning from reward omission as being potentially integrated with more widely studied phenomena involving LHb function (i.e., primary aversive conditioning and behavioral flexibility). On one hand, reward-omission experiences imbue preceding cues with properties that are functionally equivalent to those cues associated with primary aversive stimuli. On the other hand, learning in tasks requiring behavioral flexibility involves committing errors (Baker et al., 2017) and this often entails the omission of otherwise expected rewards. Therefore, we hope that understanding one of these artificially delineated fields would potentially help to elucidate some of the mechanisms involved in the others.

## Modeling the Dynamics of Acquisition and Extinction of Responding in Reward Learning

**Figure 2.**
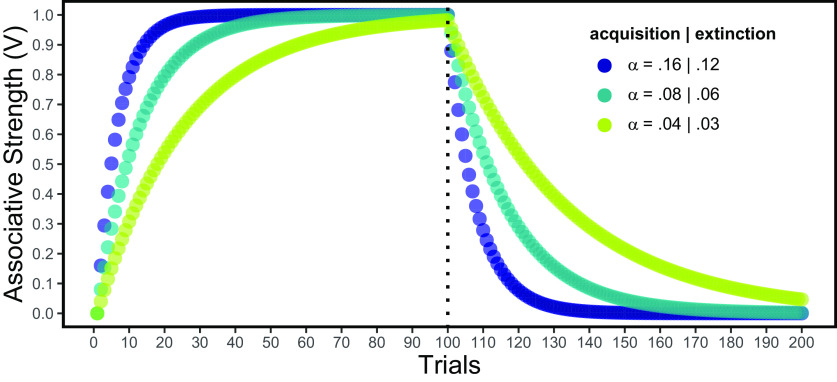
Dynamics of associative strength according to the Rescorla–Wagner model in 100 consecutive trials of acquisition (*λ* = 1) followed by 100 extinction trials (*λ* = 0). Colors represent different learning rates (*α*), assuming that extinction rates are 3/4 of acquisition rates. Note that the decrease in responding (right panel) is a dynamic process that progresses with each trial, unlike the slow and continuous decay that would be expected if we simply allow time to pass.

Learning could be roughly regarded as a process in which behavior is updated when an organism faces environmental regularities. Although learning is a continuous process (Sutton and Barto, 2018), it is sometimes methodologically and analytically useful to discretize it in trials (Harris, 2019). Trials are fractions of time in which explicit experiences are assumed to promote specific changes in future behavior. Rescorla and Wagner (1972) proposed a model accounting for the associative strength of a target stimulus given its pairings with an affectively significant event on a trial-by-trial basis. Such an associative strength or value could represent a theoretical estimation of either the vigour or the probability of responding to the target stimulus in the next trial. While “responding” was originally intended to reflect a change in overt outcome-anticipating actions, it may also represent a neural-level state change such as firing of dopamine neurons (see Roesch et al., 2012). This model states that a target stimulus gains or loses associative strength according to a learning rule. The change for this value in a given trial depends on the discrepancy of the current value of the target stimulus and that of the outcome that follows (e.g., presentation or absence of a reward). Formally:

(1) $$\Delta V_{X}=\alpha (\lambda –\Sigma V_{N}),$$

where *ΔV_X_* represents the change in the associative strength (*V*) of the target stimulus (or action; *X*) in a given trial, *λ* represents the magnitude of reward (zero if the reward is omitted), *ΣV_N_* represents the sum of the values of all cues that are present during a conditioning trial (usually, accounted for with a few of those cues), and *α,* which is bound between 0 and 1, represents a learning rate parameter. To show how the model operates, let us first consider the simplest possible example. This requires assuming that, during conditioning trials, the only relevant input besides the reward is a single cue (thus being *V_X_ ≈ ΣV_N_*). If *λ* (> 0) and *α* are held constant, *V_X_* will increase approaching *λ* throughout conditioning trials (e.g., pairings of the stimulus with reward) in a decelerated fashion, as the discrepancy between these parameters (i.e., *λ − ΣV_N_*) progressively declines (see Fig. 2, left panel). Thence, the absolute value of the parenthetical term in Equation 1 determines the amount of behavioral change on the next trial of the same type. The discrepancy between expected and experienced outcomes has been termed *prediction error* and could be regarded as a theoretical teaching signal that alters some aspect of the system to update behavior. Prediction errors can be classified as appetitive (reward-related) or aversive, and as positive or negative (Iordanova et al., 2021). In addition, prediction errors seem to dictate both Pavlovian (i.e., stimulus-outcome) and instrumental (i.e., action-outcome) learning through a homologous correction mechanism (Bouton et al., 2020; see also Eder et al., 2015).

A the physiological level, canonical midbrain dopamine neurons fire above their baseline activity upon the presentation of unexpected rewards (i.e., positive reward prediction error; *λ* > *ΣV_N_*). Such dopamine bursts increase the probability of occurrence of the actions that took place priorly, whenever the organism is presented to a similar situation in the future (i.e., positive reinforcement). In addition, dopamine bursts backpropagate to reliable signals of reward, making them acquire signaling (i.e., discrimination learning) and rewarding (i.e., conditioned reinforcement) properties in themselves (Sutton and Barto, 2018). Dopamine phasic activity (as in *V_X_* > 0) is triggered by inputs received by midbrain neurons from multiple nodes in a brain-wide network subsequent to sensory processing (Tian et al., 2016). Deceleration in the increase of associative strength with repeated experiences involving paired presentations of the stimulus and the reward would require an antagonistic mechanism. Inhibitory NAc-VTA projections have been hypothesized to play some role in said mechanism (see Mollick et al., 2020).

Once a cue or action has acquired some associative strength, the repeated omission of reward after its presentation allows for the restitution of behavior to the initial non-responsive state. Reward omissions following the presentation of an already conditioned stimulus (or whenever *λ* is smaller than Σ*V_N_;* i.e., negative reward prediction error) trigger a phasic decrease (or *dip*) in dopamine activity (Schultz et al., 1997; Matsumoto and Hikosaka, 2007). If repeated consistently, these dopamine dips are known to decrease dopamine firing to the target stimulus in subsequent trials. This translates into a decrease in both conditioned responses and reinforcing effects associated to the target stimulus until reaching a minimum value (see Fig. 2, right panel). Here, the procedure, process, and outcome are each referred to by the term “extinction^1^.” Suppression of midbrain dopamine neurons induced by LHb activity plays a key role in this (context dependent; see Footnote 1) restauration mechanism. For example, a recent study by Donaire et al. (2019) found that LHb lesions impair extinction of an appetitive response in rats. Similarly, Zapata et al. (2017) found that pharmacological inhibition of the LHb impaired extinction of responses that were previously rewarded in the presence of a stimulus. However, this effect was selective for cocaine reward and did not impair extinction of responding previously rewarded with a sucrose solution. This finding was interpreted as an indicative of the greater difficulty in withholding responses rewarded with cocaine compared with those rewarded with sucrose. Both findings reveal the participation of the LHb in decreasing previously acquired behavior when a stimulus or action is no longer followed by a reward (i.e., extinction).

## Inducing, Testing, and Modeling Net Inhibitory Effects

When conceptualizing conditioning trials as if a single cue is relevant for reward learning (such that *V_X_ = ΣV_N_*) obtaining a negative value for *V_X_*with Equation 1 is logically impossible. As *V_X_* would range from zero to *λ*, it can only vary in its degree of “rewardingness” (for lack of a better term). That is, one could conceive of a stimulus as more or less rewarding than the other, but hardly as more aversive in a general sense. However, a distinctiveness of the Rescorla–Wagner model allows to account for both net negative associative strength values and acquisition of opposed affective effects. Such feature is important because it captures aspects of key conditioning phenomena.

**Figure 3.**
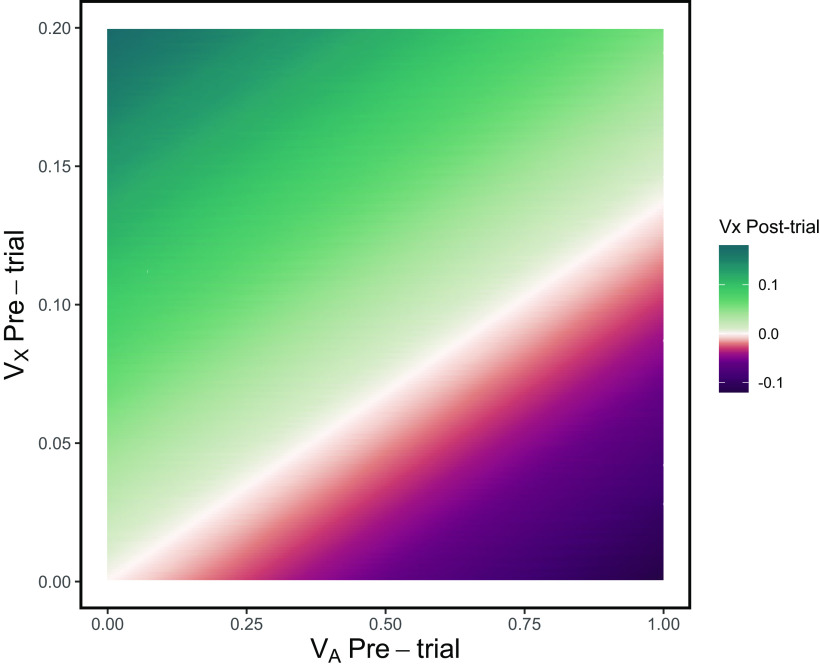
Possible values of associative strength for stimulus *X* (*V_X_*; color scale) after being presented in compound with an excitatory conditioned stimulus, *A*, and followed by the omission of reward (*λ* = 0). According to the Rescorla–Wagner model, the outcome depends on the associative strength of the companion stimulus *A* (horizontal axis) that of *X* (vertical axis) before the reward omission episode, and on learning rate (*α* = 0.12 is assumed).

A neutral stimulus that is consistently paired with the omission of an expected affectively significant event often acquires the opposite affective valence. In this case, a stimulus paired with the omission of an expected reward would acquire aversive properties (Wasserman et al., 1974). This means that the organism will be inclined to avoid it (either actively or passively) or escape from it. On the other hand, the negative summation effect occurs when a stimulus associated with the omission of reward is presented simultaneously with one that reliably predicts the reward. The usual result is that responding to the compound stimulus is reduced compared with that controlled by the reward-predicting stimulus alone (Acebes et al., 2012; Harris et al., 2014). The model of Rescorla and Wagner (1972) allows for partitioning of the elements that constitute a compound stimulus; concretely, it provides a rule to determine how associative strength values of different stimuli presented simultaneously interact trial by trial. Recall that *ΔV_X_* represents the change in the associative strength of a single element, *X*, of the assemblage of current stimuli, *N*. Whenever *ΣV_N_* exceeds the value of *λ* (as in trials involving reward omission), *V_X_* could take negative values under appropriate conditions. In order for *X* to end up with a net negative associative strength following reward omission requires that (1) its current associative strength to be sufficiently low and (2) the remaining of the coextensive stimuli have some associative value (see Fig. 3).

In the Rescorla–Wagner model, the vigour of responses evoked by *ΣV_N_* is assumed to be a sort of algebraic sum of the values of current stimuli (obviating biological limits). Crucially, this model assumes that the context (cues that are continually present) could acquire associative strength and is thus capable of meaningfully impacting learning. For example, presenting *X* without the reward and the reward without *X* with time intervals in between would imbue that stimulus with net inhibitory properties (i.e., *V_X_ < 0*). This protocol is known as the *explicitly unpaired procedure*. In such a condition, the context, *C*, acquires a positive associative strength (*V_C_* > 0), by being present whenever the reward is delivered. Importantly, the context is also present during the trials involving the *X* stimulus plus the omission of reward, being *ΣV_N_* equal to *V_C_* + *V_X_* (Wagner and Rescorla, 1972). Then, *ΔV_X_* will be negative for every such trial, accruing a negative value progressively over *V_X_*. This would manifest as aversion-related behaviors toward *X* (see Wasserman et al., 1974) and as a subtraction of the response vigour evoked by a reward-predicting stimulus whenever *X* is presented (i.e., $X\in \Sigma V_{N}\;<\;X\notin \Sigma V_{N}$). Such outcomes are justified by two assumptions. First, a negative *V_X_* implies a subtraction of the response-evoking potential of concurrent excitatory stimuli (Wagner and Rescorla, 1972). Second, negative associative values imply that a stimulus has an affective valence that opposes that of the outcome with which it was trained (Daly and Daly, 1982).

**Figure 4.**
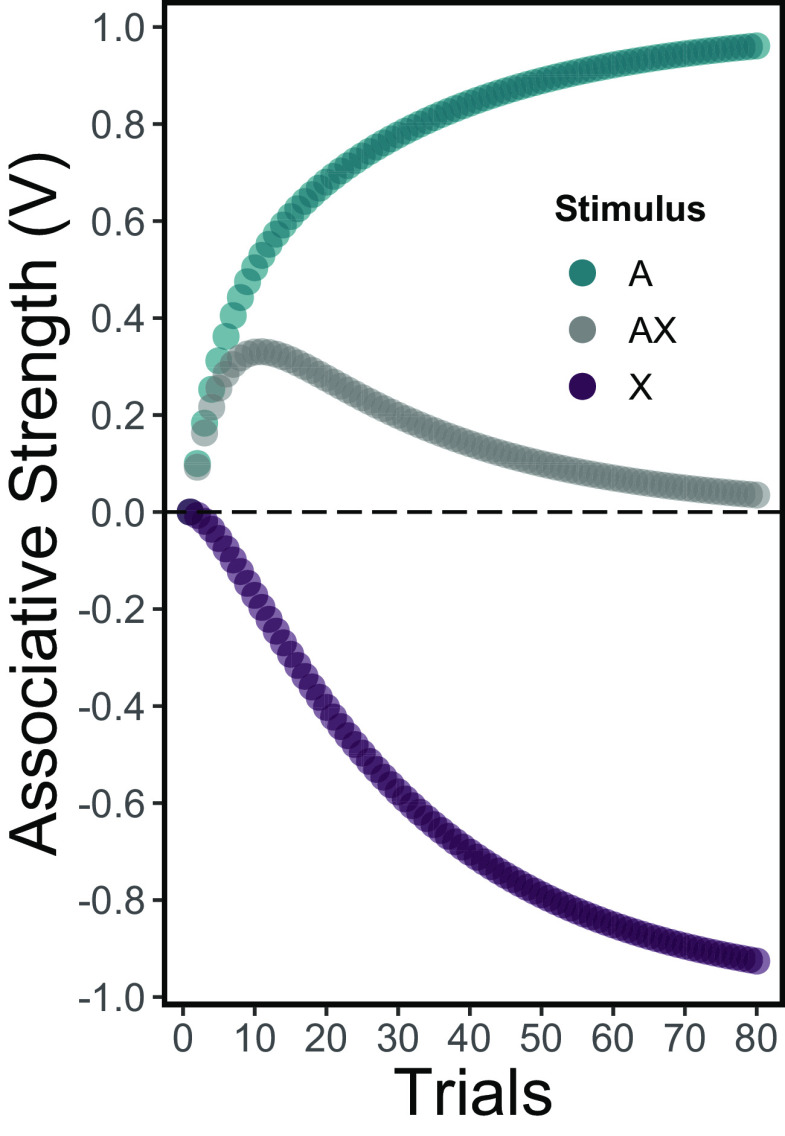
Associative strength, according to the Rescorla–Wagner model, of stimuli involved in a feature-negative discrimination protocol in 80 trials of each type. Trials of the stimulus *A* plus reward and not rewarded *A-with-X* trials were assumed to alternate non-randomly. Values of *α* were set at 0.1 and 0.075 for rewarded (*λ* = 1) and not rewarded (*λ* = 0) trials, respectively. The associative strength of the stimulus *A* and the compound stimulus *AX* could be observed in actual performance. In contrast, the associative strength of *X* alone (i.e., the conditioned inhibitor) is theoretically inferred from the model and could be revealed by special tests (see text).

A convenient way to conceptually and empirically instantiate these attributes of the Rescorla–Wagner model is the *feature-negative discrimination protocol*^2^. This technique consists in presenting two types of conditioning trials, usually in a random fashion. One type of trial consists of presenting a single stimulus, *A*, followed by an affective outcome (e.g., reward). The remaining type of trial consists of the same stimulus accompanied by another stimulus, *X*, followed by the omission of that outcome. Such a manipulation, according to the Rescorla–Wagner model, leads stimulus *A* to acquire a positive associative strength and stimulus *X* to acquire a deep negative associative strength (see Fig. 4). This hypothetical attribute is known as *conditioned inhibition*, and Rescorla (1969) stated that it should be empirically demonstrated using two special tests. One of these tests is the above-described *negative summation* effect and the other is the *retardation in the acquisition* of a conditioned response by the target stimulus. Also, as stated above, conditioned inhibitors trained with an outcome of a particular affective valence have been documented to acquire an opposed valence (i.e., appetitive to aversive and vice versa).

## Dopamine Dips Propagate from Reward Omission to Events That Predict It

The omission of an expected reward seems to promote a similar backpropagation process to the one that takes place when a cue predicts reward. Interestingly, the LHb is likely to play a crucial role in this process (see also Mollick et al., 2020). As mentioned above, reward omission triggers a phasic depression in dopamine release. In turn, this promotes a decrease in dopamine release (and its behavioral outcomes) toward cues that predict reward omissions. This implies that dopamine dips would promote a plastic change in the behaving agent which is opposite to that involved in reward. The clearest illustrations of this claim are brought by two complementary experiments to be described below.

First, Tobler et al. (2003) exposed macaque monkeys to a feature-negative discrimination protocol (see section above). In this study, the presentation of a cue alone predicted reward delivery. The presentation of the same cue accompanied by another stimulus, the conditioned inhibitor, predicted reward omission. Through training, the conditioned inhibitor acquired the capacity of counteracting the effects of the companion cue in reward omission trials (see Fig. 4, gray circles). This tendency was also observed when the conditioned inhibitor was presented along with a different reward-predicting cue in a novel compound (i.e., negative summation). Notably, those effects were reported at both behavioral (licking response) and neural (dopamine neuron activity) levels of observation. Most importantly, the presentation of the conditioned inhibitor alone, while behaviorally silent, was capable of decreasing baseline dopamine firing levels. This revealed that reward omission promotes a physiological transfer of function from the situation in which the reward is actually omitted to the cue that consistently predicts it.

The second experiment was conducted recently by Chang et al. (2018), using rats as subjects. In this study, brief dopamine dips were artificially induced in midbrain neurons following the simultaneous presentation of a reward-predicting cue and a novel cue. After training, the novel cue acquired the properties of a conditioned inhibitor, as per the conventional tests of negative summation and retardation of acquisition. Crucially, in the training trials in which this novel cue was involved the reward was *not* omitted. Thus, the inhibitory effects in the target cue must have arisen from the artificial dips in dopamine firing, complementing the findings of Tobler et al. (2003). On one hand, Tobler et al. (2003) demonstrated that dopamine dips could propagate from the omission of reward to the presentation of an arbitrary event; on the other hand, Chang et al. (2018) demonstrated that the dopamine dips themselves are sufficient for this to occur.

As we mentioned above, one of the most robust physiological effects of LHb activation is producing phasic dips in dopamine firing. Therefore, both Tobler et al. (2003)’s and Chang et al. (2018)’s findings are relevant to elucidate the function of LHb physiology in learning from experiences involving reward omission. In another recent study, Mollick et al. (2021) intended to test this notion with humans in an fMRI study. The aim of this study was to replicate Tobler et al. (2003)’s behavioral protocol but including the measurement of the activity in different regions across the whole brain (not only in midbrain dopamine cells). Mollick et al. (2021) indeed captured greater LHb activity during the presentation of a conditioned inhibitor compared with control stimuli; however, this difference did not survive correction for multiple comparisons. Therefore, the authors recommend further replication before any inference can be made. While an earlier human fMRI study (Salas et al., 2010) already documented that the habenula is activated when an expected reward is omitted (i.e., negative reward prediction error), evidence for the backpropagation of this effect remains elusive. A possible explanation for the null result reported by Mollick et al. (2021) is that the human LHb is quite small and cannot be differentiated from the medial habenula with standard fMRI resolutions (cf. Salas et al., 2010). Similarly, the study by Mollick et al. (2021) failed to capture decreased activity in midbrain dopamine regions during reward omissions. The authors argued that their fMRI spatial resolution may have also precluded the distinction between these regions and the adjacent GABAergic RTMg (which was presumably active as well during reward omissions).

## Concurrent Reward Cues Contribute to Dopamine Dips and Their Propagation

The above section concluded that dopamine dips may function as “teaching” signals for gradually imbuing a stimulus with negative associative strength. The Rescorla–Wagner model states that the negative associative strength accrued by a stimulus that is paired with reward omission depends on concurrent reward cues (see Fig. 3). Therefore, if we assume that dopamine dips depend on LHb activation, the latter would rely on current information about an impending reward. That is, if a stimulus is followed by the omission of reward, its associative strength would not change unless it is coextensive with reward-related cues. Therefore, LHb activation might not be induced by the sheer non-occurrence of reward. Rather, the LHb may become active when this non-occurrence coincides with signals from other brain regions indicating probable reward. Furthermore, the greater the signal for reward the larger the downshift in associative strength of (and the aversion imbued to) the stimulus paired with reward omission (see Fig. 3). Accordingly, dopamine dips induced by reward omission would be proportional to the probability (or magnitude) of the reward associated with a conditioned stimulus. Tian and Uchida (2015) found evidence supporting this assumption using mice as subjects and three different probabilities of reward associated with different target stimuli. Crucially, this pattern of results was hindered in animals with bilateral lesions in the whole habenular complex.

A relevant consideration is the origin of the signals required for invigorating the LHb activity on signals of an impending reward. The entopeduncular nucleus (EPN; border region of the globus pallidus internal segment in primates; Hong and Hikosaka, 2008) and lateral hypothalamus (LH; Stamatakis et al., 2016) provide excitatory inputs to the LHb. However, up to this point, it remains unclear how impending reward signals mediate the invigoration of the LHb. An elegant study with macaques by Hong and Hikosaka (2013) found that electric stimulation of the ventral pallidum (VP) consistently inhibited the activity of the LHb. These authors conjectured that an interaction between inputs from the EPN and the VP in the LHb was responsible for the activation of the LHb. Recent evidence indicates that GABAergic neurons in the VP are activated by surprising rewards and reward-predicting cues and inhibited by aversive stimuli and reward omission (Wheeler and Carelli, 2006; Stephenson-Jones et al., 2020). Crucially, inhibition of LHb projecting VP GABAergic neurons on reward omission depends on current reward cues. In short, these VP neurons showed a biphasic ascending→descending pattern of activation to the reward-cue→reward-omission sequence. Therefore, this structure is a strong candidate for providing signals to invigorate the activation of the LHb in reward omission episodes via disinhibition.

The strength with which the EPN triggers LHb activation may depend on the prior tone of VP inhibitory inputs. In fact, Stephenson-Jones et al. (2020) reported that a number of GABAergic VP neurons synapsing with the LHb exhibited a sustained pattern of activation during reward-predicting cues. Then, sudden inhibition of these neurons upon reward omission may combine with activation of EPN signals for strongly disinhibiting the LHb. Stephenson-Jones et al. (2020) also observed that a small subpopulation of LHb-projecting glutamatergic VP neurons showed the opposite pattern of activation to that of GABAergic ones. Activation of these neurons may add up to complement the potent disinhibition-excitation process in the LHb during omission of an expected reward.

Knowing the source of excitatory and inhibitory inputs to the LHb is an essential matter. However, this leads to the task of identifying the upstream inputs of these sources (Bromberg-Martin et al., 2010) and so on. Hong and Hikosaka (2013) speculated that the EPN may activate the LHb through disinhibitory inputs from striosomal regions of the dorsal striatum. This conjecture has recently been supported by a study conducted by Hong et al. (2019), in which the activation of striatal neurons inside or nearby striosomes correlated with activation of the LHb. Hong et al. (2019) further advanced the hypothesis that striosomes may convey both excitatory and inhibitory inputs to the LHb. Regarding the VP, Stephenson-Jones et al. (2020) reported that neurons in this structure targeting the LHb became active in a state-dependent fashion; specifically, these neurons fire according to the motivational status of the subjects, which is probably mediated by hypothalamic signals. It seems reasonable that activation of the LHb would be dependent on the energetic supply status or sexual drive at a particular moment. In short, the omission of reward should not cause substantial disturbance to a sated individual. This could be another possibility for explaining the failure of Mollick et al. (2021) to replicate Tobler et al. (2003)’s findings. Both studies used fruit juice as reward. However, in the latter study macaques were liquid deprived, while in the former participants were recruited based on self-reported preference for the type of juice employed. The motivational state of participants in Mollick et al. (2021)’s study was probably insufficient to induce strong physiological responses in the target regions.

## The LHb Promotes Stimulus-Specific and Response-Specific Inhibitory Effects

Although feature-negative discrimination is a prototypic procedure for inducing conditioned inhibition effects in appetitive settings (which here we are proposing as a behavioral phenotypic model of LHb function), other protocols are also capable of doing so (see Savastano et al., 1999). Likewise, other behavioral manifestations, besides negative summation and retardation of acquisition tests, could be used to certify conditioned inhibition (Wasserman et al., 1974). For example, in a pivotal study, Laurent et al. (2017) exposed rats to a backward conditioning preparation to induce conditioned inhibition. Then, conditioned inhibitory properties of stimuli were tested through a Pavlovian-to-instrumental transfer (PIT) test. This backward conditioning procedure consisted in presenting one of two different auditory target stimuli following, rather than preceding, as typically done, the presentation of two different rewards; each target stimuli was consistently associated with its corresponding reward in a backward fashion (reward→stimulus). Next, rats pressed two levers to obtain rewards and each of the rewards used in the previous phase was associated with a different lever. In the PIT test, the levers were present but pressing was no longer followed by reward. In that condition, the target stimuli that were used in the first phase were presented randomly and lever pressing was recorded. Conditioned inhibition was revealed inasmuch each target stimulus biased lever-pressing toward the opposite lever than that associated with its corresponding reward. Importantly, Laurent et al. (2017) found that this outcome was impaired by bilateral lesions of the LHb.

The disruption of conditioned inhibition was first observed by performing an electrolytic lesion in the entire LHb. This result was replicated when the authors induced a selective ablation of neurons in the LHb that projected to the RMTg via a viral technique. Therefore, the authors concluded that this LHb-RMTg pathway is crucial for learning these specific negative predictions about rewards. Remarkably, electrolytic lesions in the LHb did not disrupt PIT performance for rats trained with traditional forward-conditioning (stimulus→reward) procedure. This suggests that the disruptive effect was confined to inhibitory learning (see also Zapata et al., 2017). Laurent et al. (2017)’s findings provide important insights for understanding the role of LHb-RMTg pathway in the development of conditioned inhibition in appetitive settings. Beyond generally impairing responding when reward is negatively predicted, the LHb seems to promote learning to perform specific action patterns to specific cues. Apparently, this structure participates in enabling alternative behaviors for exploiting alternative resources in the environment when the omission of a particular reward is imminent.

Notably, the findings reported by Laurent et al. (2017) challenge the Rescorla–Wagner model. According to this model, a stimulus that is explicitly unpaired with a reward can acquire a negative associative strength (i.e., become a conditioned inhibitor). This would be mediated by the relatively high associative strength in the context that coextends with the target stimulus preceding reward omission. The Rescorla–Wagner model predicts that backward conditioning would lead as well to conditioned inhibition by this rationale (see Wagner and Rescorla, 1972). In backward conditioning, the target stimulus also co-occurs with the presumably excitatory context. However, Laurent et al. (2017) observed specific inhibitory effects for each of the target stimuli in their study. It is worth stressing that those target stimuli were trained at the same time range and in the same context. Thus, the target stimuli presumably acquired conditioned inhibitory properties by being (negatively) associated with each reward. In contrast, the Rescorla–Wagner model predicts that both target stimuli would acquire general inhibitory properties. This follows from the rationale that each target stimulus was paired with the absence of either reward in a context associated with both.

At the behavioral level, Laurent et al. (2017)’s findings can be readily accounted for by the sometimes-opponent-process (SOP) model (see Vogel et al., 2019). This associative learning model invokes stimuli after-effects that are capable of shaping behavior if they are appropriately paired with other events. According to the SOP model, there is an inhibitory (opponent) decaying trace of its own kind following the delivery of each particular reward. In the study of Laurent et al. (2017), each reward’s unique trace would have been paired with each of the target stimuli. As a consequence, each target stimulus acquired reward-specific conditioned inhibitory properties. As LHb lesions completely abolished the behavioral effect resulting from this protocol, this structure appears to play a crucial role in this phenomenon. However, the putative physiological means by which LHb mediates learning in conditions such as those in Laurent et al. (2017)’s study remain to be determined.

## The LHb Participates in Inhibitory Learning Even without Temporally Specific Reward Omission

The findings reported by Laurent et al. (2017) merit further examination. Based on this study, we can assume that the LHb could exert its effects without acute episodes involving the omission of an expected reward. Such a process is also instantiated in explicitly unpaired procedures, in which a target stimulus acquires inhibitory properties solely by alternating with reward. A recent study by Choi et al. (2020) sought to test the hypothesis that the LHb is involved in learning the association of a cue with the absence of reward. These authors exposed rats to an explicitly unpaired conditioning procedure that used a light as a negative predictor of food delivery. Choi et al. (2020) found increased c-Fos expression in the LHb of rats exposed to an explicitly unpaired conditioning procedure compared to controls. This result supports the idea that the LHb is engaged in the learning process that takes place when a stimulus signals the absence of reward. In a separate experiment, these authors did not find any disruption in performance on this protocol when they induced excitotoxic LHb lesions. However, this should not be taken as negative evidence of the involvement of the LHb in inhibitory learning. This finding may be explained on the basis of the need for special tests (e.g., summation, retardation, PIT; see Rescorla, 1969) to certify that an inhibitory stimulus is capable to counteract reward-related behaviors. Unfortunately, Choi et al. (2020) did not conduct any of the available tests for assessing conditioned inhibition.

Both in backward conditioning and in explicitly unpaired conditioning preparations, the reward would not be expected to occur in any particular moment; specifically, a reward delivery is scheduled to occur at random intervals without discrete cues anticipating it, unlike in extinction and in feature-negative discrimination procedures. The ability of the LHb to modulate reward seeking, even in paradigms not involving explicit reward omissions, raise the question of what the mechanisms involved are. A recent study by Wang et al. (2017) may shed light in this issue. These authors described a rebound excitation in the LHb following the inhibition typically produced by reward delivery. Given appropriate timing, the traces of such a rebound excitation could have been paired with the target stimuli for the control, but not for the lesioned, subjects in Laurent et al. (2017)’s study. Such pairings would facilitate the propagation of LHb excitation, presumably promoting dopamine suppression, eventually on the target stimuli alone. However, this mechanism cannot account readily for inhibitory learning in explicitly unpaired conditioning procedures. In these protocols, the target stimulus does not consistently follow the reward, but these events are rather separated by intertrial intervals of varying durations. Therefore, decreases in dopamine mediated by rebound activity of the LHb could hardly explain the inhibitory properties acquired by a target stimulus in explicitly unpaired procedures.

The LHb is known to modulate other monoamines besides dopamine, such as serotonin (Amat et al., 2001). Tonic serotoninergic activity depends on accumulated experience with rewards (Cohen et al., 2015). In turn, serotonergic tone determines the effects of phasic activity in serotonergic neurons, most of which is known to occur during aversive experiences (Cools et al., 2011). Thus, the LHb may participate in accumulating information about reward probability in a given environment. This would tune serotonin levels during inter-reward intervals on explicitly unpaired procedures, which might determine the affectiveness of salient cues throughout those intervals. A similar effect may also be prompted by the LHb via modulation of tonic dopamine levels (see Lecourtier et al., 2008), which would not contradict with the serotonergic hypothesis. However, it is yet to be determined with certainty whether the LHb participates in inhibitory learning from explicitly unpaired protocols. This will require using either *in vivo* real-time recording or special tests for conditioned inhibition in lesion/inactivation studies. In addition, it is not clear how the LHb would be more active in explicitly unpaired conditions than in control conditions involving equivalent reward density, which was the main result reported by Choi et al. (2020). A possibility is that the LHb is relatively inactive when a reward is fully predicted by a cue. Conversely, it may be tonically active in ambiguous conditions; for example, when the reward or the cue occur at any given time without notice (i.e., are explicitly unpaired).

## The Habenulo-Meso-Cortical Excitatory Pathway May Play a Role in Negative Reward Prediction Error

The glutamatergic LHb-VTA-mPFC pathway (see Fig. 1) is less well-known than the LHb-RMTg pathway but may also play an important role in inhibitory learning from reward omission. As we described earlier, unlike other LHb efferents this pathway promotes, rather than inhibiting, dopaminergic transmission. Perhaps, the most crucial evidence supporting this idea stems from a study conducted by Lammel et al. (2012). These authors reported that blocking dopaminergic transmission in the mPFC abolishes the capacity of the LHb to generate aversion to spatial stimuli. Therefore, this pathway may be crucial in complementing dips in meso-striatal dopaminergic release for learning from negative reward prediction errors. Two putative, not mutually exclusive, mechanisms for such an effect could be (1) directly opposing midbrain dopamine activity (see Jo et al., 2013) and (2) selecting which stimuli should be filtered to control behavior (see Vander Weele et al., 2018). The connectivity relationship between the mPFC and the LHb is reciprocal (see Mathis et al., 2017) and their afferences also converge in certain brain locations (Varga et al., 2003). The mPFC has long been considered a key brain region for restraining inappropriate actions and adjusting behavior when errors occur (Ragozzino et al., 1999) but only recently this has been considered from an associative learning perspective. Although research on LHb-mPFC interaction is still scant, we outline some ideas on how these regions may jointly contribute to learning from negative reward prediction errors.

We should consider first that the mPFC is divided in functionally dissociable anatomic subregions, at least for some eutherian mammals (see Ongur and Price, 2000). A subregion that might play a role in the LHb’s network is the prelimbic cortex of rodents, which presumably corresponds to the pregenual anterior cingulate in primates (see Laubach et al., 2018). Several studies have linked this subregion with the ability of mammals to restrain a dominant (previously rewarded) response (Laubach et al., 2015). An outstanding example of this is a study conducted by Meyer and Bucci (2014) using rats as subjects. These authors found that prelimbic, but not in the adjacent infralimbic, mPFC lesions impaired inhibitory learning process in an appetitive feature-negative discrimination paradigm in rats (i.e., differentiation in responding to A and AX in Fig. 4). As we described above, this paradigm consists in presenting a target stimulus that signals reward omission. However, importantly, this target stimulus occurs in the presence of a cue that otherwise consistently predicts reward delivery. To effectively learn how to stop reward-related responses in such situation, subjects must solve the conflict between reward and reward-omission cues that are presented simultaneously.

It has been suggested that the opposing effects of the prelimbic division of the mPFC upon the reward systems is exerted via an aversion mechanism. This rationale is supported by evidence that this region is involved in fear learning (Burgos-Robles et al., 2009; Piantadosi et al., 2020) and exhibits robust excitatory connections with the basal amygdala (Sotres-Bayon et al., 2012). Conversely, the infralimbic portion of the mPFC, has been related to both the expression of habitual reward-related responses (Haddon and Killcross, 2011) and fear suppression (Santini et al., 2008; but see Capuzzo and Floresco, 2020). The involvement of the infralimbic cortex in the latter of these processes could be accounted for in terms of mediation of a subjective relief state. Such a state would be functionally equivalent to reward and, therefore, opposed to aversive states in a hierarchical fashion under appropriate circumstances.

However, some findings (e.g., Sharpe and Killcross, 2015) seem to contradict this prelimbic–aversive and infralimbic–appetitive notion, which prompts deviations from this theoretical scheme. Instead of the aversive–appetitive dichotomy, Sharpe and Killcross (2018) sustain that the infralimbic cortex promotes attention to stimuli that reliably predict affectively significant events, regardless of their valence (i.e., reflective control). In contrast, the prelimbic cortex would promote attention to higher order cues setting hierarchical (as in conditional probabilities) relationships between stimuli and relevant outcomes (i.e., proactive control), again, regardless of their affective valence. A similar point has been raised by Hardung et al. (2017), who found evidence for a functional gradient in the mPFC spanning from the prelimbic to the lateral-orbital cortex (passing through the infralimbic, medial-orbital and ventral-orbital regions). These authors reported that regions near to the prelimbic cortex tend to participate more in proactive control, while regions near the lateral-orbital cortex participate more in reflective control. However, this study was conducted in an appetitive setting, so hierarchical control (reactive vs proactive) and opponent affective control (appetitive vs aversive) hypotheses cannot be disambiguated. While cortical inputs to the LHb are generally modest, the prelimbic cortex makes the largest contribution among the regions of the cortex innervating this structure (Yetnikoff et al., 2015). This may indicate that this pathway serves as an either positive or negative feedback mechanism, depending on whether these connections are excitatory or inhibitory. Being the LHb a major aversive center, the former possibility would be at odds with the approach of Sharpe and Killcross (2018).

Furthermore, a recent finding has tilted the scale in favor of the aversive–appetitive modularity of the mPFC. Yan et al. (2019) reported that spiking in a subpopulation of neurons in rats dorsomedial PFC (dmPFC; including prelimbic and dorsal cingulate cortex) was (1) increased upon the presentation of a cue signaling an imminent electric shock, and (2) increased, but considerably less, and then decayed below baseline on the presentation of a cue that signals the omission of the shock. In addition, Yan et al. (2019) showed that the cue signaling shock omission passed the tests of summation and retardation for conditioned inhibition, both at neural and behavioral levels. Interestingly, this pattern of results mirrors those in the study by Tobler et al. (2003) in an appetitive setting with macaques. In brief, suppressing fear responses involves inhibition of dmPFC neurons, suggesting that this region serves primarily to facilitate aversive learning rather than having a general hierarchical control function. This is in line with the idea that LHb input to the prelimbic cortex may counteract the effects of reward cues via an opponent affective process. Such a mechanism has been regarded as one of the necessary conditions for the occurrence of inhibitory learning and performance (see Konorski, 1948; Pearce and Hall, 1980). However, this mechanism may be complemented with others, such as threshold increase and attention to informative cues (Laubach et al., 2015).

## Relevance of the LHb for Mental Health

**Figure 5.**
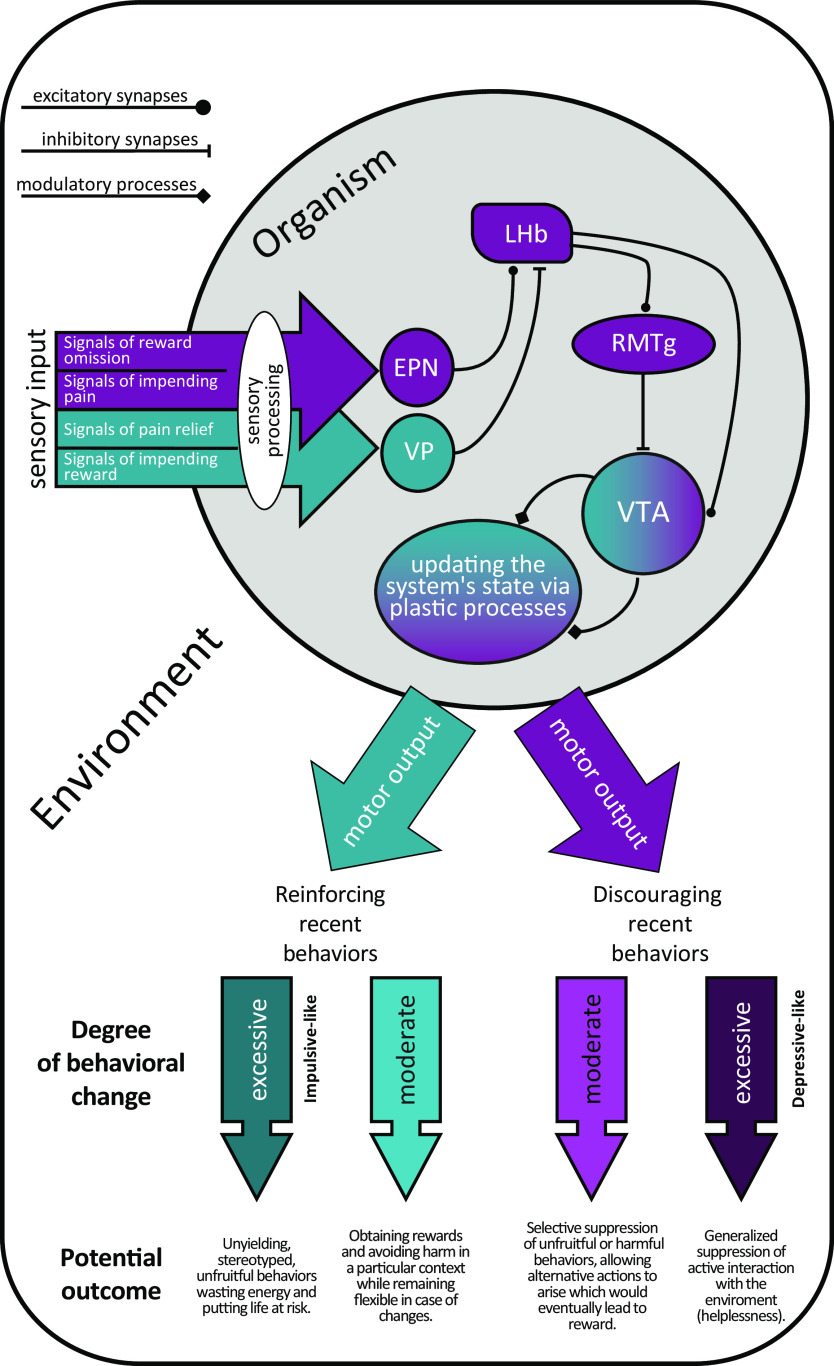
Simplified diagram representing the participation of the LHb in plastic brain processes for adaptively updating behavior and hypothesized consequences of excessive activity (dark purple) or inactivity (dark aqua) of this brain structure.

There is an emerging literature suggesting that many psychiatric disorders can be characterized as alterations of the ability to predict rewards and aversive outcomes (Byrom and Murphy, 2018). Therefore, human wellbeing could be understood, at least in part, in terms of the dynamics of associative learning and outcome prediction (Papalini et al., 2020). Thus, disrupting the neural underpinnings of outcome predictions, either in an upward or downward direction, could lead to maladaptive behaviors that threaten people’s subjective wellbeing. Disappointment, frustration, and discomfort stemming from worse than expected outcomes are useful for directing behavior to adaptive ways of interaction with our surroundings (see Fig. 5, light purple arrows). However, if these subjective sensations are lacking or excessive, it is likely that problematic behaviors will arise (see Fig. 5, dark colored arrows).

Some authors have proposed that a pervasive reward learning process (Chow et al., 2017; Sosa and dos Santos, 2019a) can explain maladaptive decision-making in impulsive and risk-taking behaviors. This may arise in part from a deficit in learning from negative reward prediction errors (Laude et al., 2014; Sosa and dos Santos, 2019b; see Fig. 5, dark aqua arrow). Impulsive-like behavior is often studied in choice protocols with animal models. A study by Stopper and Floresco (2014) showed that LHb inactivation led to indifference when rats choose between ambiguous alternatives. Specifically, subjects were biased away from the most profitable of two alternative rewards when a delay was imposed between the selecting action and that reward; however, this bias was abolished by LHb inactivation. This suggests that delay aversion might stem from a negative reward prediction error mechanism, rendering the puzzle even more complex. On the other hand, studies involving humans and rodent models of helplessness (Gold and Kadriu, 2019; Jakobs et al., 2019) have suggested that an over-reactivity of the LHb can induce symptoms associated with depression (see Fig. 5, dark purple arrows). Such symptoms include reluctance to explore new environments and unwillingness to initiate challenging actions, which could be interpreted as a constant state of disappointment or sense of doom (Proulx et al., 2014). In that case, atypically high reward expectations could lead to a pervasive and maladaptive enhanced inhibitory learning. Another possibility is that some individuals may have a proclivity to overgeneralize learning from negative reward prediction errors, which would cause a decreased activity overall (a major symptom of depression). These putative mechanisms are, of course, not mutually exclusive.

Synthetizing behavioral and neurophysiological studies could serve to address the etiology and phenomenology of these psychiatric conditions. Such an enterprise would require integrating details regarding behavioral changes in response to environmental constraints and the underlying neurobiological mechanisms involved. This is precisely what we have attempted in the present article. If ongoing research continues to make advances, then this perspective could serve to guide clinical practice. An example would be determining whether psychotherapy would be enough to tackle a particular case or whether combining it with pharmacotherapy would be pertinent. Knowledge about neural and behavioral dynamics might serve to design combined intensive interventions to avoid long-term side effects of medications and dependency (Hengartner et al., 2019). Additionally, understanding morphogenesis and wiring of the LHb during embryological development could be relevant to inquire whether disturbances in these processes underlie problematic behavioral traits (Schmidt and Pasterkamp, 2017).

Some recent studies have furthered our understanding of behavioral-physiological interactions involving the LHb from basic science to application. For example, Zhu et al. (2019) found an association between subclinical depression and atypical connections in the dorsal posterior thalamus and the LHb. This peculiar connectivity of the LHb might predispose people to depression or, alternatively, some life events could promote behavioral patterns leading to that phenotype. Experiences of reward loss could vary regarding the time frame and magnitude of the involved rewards (Papini et al., 2006). A person could lose money during a gambling night with friends, which could be a relatively innocuous experience, or lose a lifetime partner, being a devastating event. Significant life experiences can cause detectable changes in brain networks (Matthews et al., 2016) and tracking these changes can lead to finding ways to understand the underpinnings of behavioral phenotypes associated with psychiatric conditions. An intriguing example is that attenuating LHb activity has been shown to ameliorate depressive-like symptoms induced by maternal separation in mice (Tchenio et al., 2017).

## Directions for Moving the Field Forward

Computational modeling of associative phenomena is just one possible approach to account for the relationship between LHb physiology and inhibitory learning. There are other alternatives for improving our understanding of this topic, even in a more detailed manner, such as neural network models. These are powerful tools for envisaging plausible mechanisms implemented by biological agents to interact adaptively with their environment. A clear advantage of these models is that they make physiological hypotheses more tenable (Frank, 2005). To our knowledge, only few recently proposed neural-network models (see Vitay and Hamker, 2014; Mollick et al., 2020) have incorporated nodes regarding the physiology of the LHb. Even so, those models do not account for the multifaceted circuitry (e.g., including the LHb-VTA-mPFC pathway) of this structure which, we argue, is highly relevant for an utter understanding of its role in inhibitory learning. Lack of inclusion of the LHb into neural network modeling (Collins and Frank, 2014; Burgos and García-Leal, 2015; Sutton and Barto, 2018; O’Reilly et al., 2019) may be partly because of an overemphasis in performance over learning of inhibitory control. Cortical-thalamic-striatal circuits are often invoked to account for inhibitory performance, often without formally specifying how outcomes shape future actions (Verbruggen et al., 2014; van Wijk et al., 2020). An integration of learning and performance models should be pursued to increase our understanding of the broad network in which the LHb participates.

Another matter for future consideration is whether the role of the LHb in learning from reward omission is exclusive for edible rewards or it extends to other types of reward, such as sexual stimuli for receptive individuals. In addition, our understanding of the behavioral neuroscience regarding the LHb come from select model species. It has been hypothesized that the habenular complex evolved in an ancestor of vertebrates to enable circadian-determined movement modulation (Hikosaka, 2010). In this sense, the LHb may have later evolved its role for suppressing specific actions in response to more dynamic environmental information. Such exaptation hypothesis implies that the habenula of basal lineages did not play the same role it does in extant vertebrates. That, in turn, implies that either basal vertebrate lineages could not learn from reward omission or that they did so by recruiting other mechanisms.

Even invertebrates possess mechanisms for learning from situations involving negative prediction errors (i.e., conditioned inhibition; Britton and Farley, 1999; Couvillon et al., 2003; Acebes et al., 2012; Durrieu et al., 2020). Although this fact is appealing, for now it is fairly limited to the behavioral level of observation. However, an emerging field of research is currently scrutinizing the neural underpinnings of negative reward prediction errors in *Drosophila* flies (Felsenberg et al., 2017). In these animals, a bilateral structure known as the mushroom body supports associative learning using dopamine as a plasticity factor, in a similar fashion as the vertebrate brain does (Aso et al., 2014). The mushroom body possesses different subdivisions, which selectively release dopamine during reward or aversive stimuli. Intriguingly, Felsenberg et al. (2017) reported that inactivating aversion-related dopamine neurons preclude the bias in behavior otherwise produced by pairing a stimulus with reward omission. It has been suggested that aversive dopamine neurons directly oppose to reward-related dopamine neurons in the mushroom body (Perisse et al., 2016). This may indicate that invertebrate nervous systems possess the hierarchical architecture necessary for inhibitory learning, such as that found in vertebrates. Remarkably, these diverging nervous systems also exhibit antagonistic dopaminergic subsystems, similar to those that the LHb orchestrate in the vertebrate brain. Future research in this field may uncover the evolutionary origins of negative reward prediction errors and a more precise dating of the origins and precursors of the LHb.

## Summary and Concluding Remarks

Organisms benefit from possessing mechanisms to track resources in their surroundings and adjust their actions to obtain maximum profit. This relies on a delicate balance between responding and withholding specific responses whenever it is appropriate. Sensorimotor feedforward loops are mediated by long-term potentiation in some locations of the striatum induced by midbrain dopaminergic inputs (Yagishita et al., 2014). Conversely, there are several processes that prevent spurious sensorimotor loops, which would potentially waste energy and put the organism at risk. A convenient paradigm to study one such process is conditioned inhibition, a subclass of Pavlovian learning phenomena. This paradigm could serve as a principled and physiologically informed behavioral phenotype model of negative error prediction. Negative feedback control mechanisms had been recently proposed to be of primary importance to understand adaptive behavior (Yin, 2020). Therefore, conditioned inhibition might be a particularly relevant and timely conceptual and methodological tool. Intriguingly, although inhibitory learning has been thought to be multifaceted in nature (Sosa and Ramírez, 2019), several of its manifestations seem to implicate the LHb.

Omission of expected rewards at a precise time promotes dopamine dips in canonical midbrain neurons with a remarkable contribution of the LHb (see Mollick et al., 2020). These dopamine dips back-propagate to stimuli that consistently predict reward omission in a way that resembles plastic changes associated with dopamine release (Tobler et al., 2003). All things being equal, dopamine dips can counteract phasic dopamine effects on behavior (Chang et al., 2018). This accounts for LHb’s contribution to extinction of a previously acquired response (Zapata et al., 2017), feature negative discrimination, negative summation, and retardation of acquisition in appetitive settings (Tobler et al., 2003). However, although the activity of the LHb is associated with dopamine dips, it may also participate in learning processes beyond general suppression of previously rewarded actions (Laurent et al., 2017). This selective effect is remarkable given that other brain areas have been associated with global response inhibition (see Wiecki and Frank, 2013).

Even if LHb’s suppressing effects on midbrain dopamine neurons are robust, the excitatory LHb-VTA-mPFC pathway may also play a role in inhibitory learning from reward omission. Lesions of the prelimbic portion of the mPFC impair inhibitory learning in appetitive settings (Meyer and Bucci, 2014). This region receives indirect dopaminergic input from the LHb and its activation has been implicated in aversion learning (Lammel et al., 2012). Some learning theories have proposed that conditioned inhibition is partly determined by antagonizing the effect of an affectively loaded conditioned stimulus (Konorski, 1948; Pearce and Hall, 1980). The activity of this pathway leads to dopamine release in the mPFC, which may allow channeling relevant sensory inputs to key plastic brain regions (Vander Weele et al., 2018). Therefore, interactions of the LHb with aversive centers via the prelimbic cortex may facilitate imbuing cues with the capacity of counteracting reward-seeking tendencies. Disrupting either the excitatory or inhibitory LHb pathways has been shown to hamper inhibitory learning processes to some degree. Whether and how those pathways may interact or complement each other to promote inhibitory learning remains a matter of further investigation.

Feature-negative discrimination and extinction procedures promote a decrease (or even net negative values) in associative strength, presumably through the omission of a reward at a precise moment following a cue. However, backward conditioning also induces conditioned inhibition and this effect is impaired by specific ablation of the LHb-RMTg pathway (Laurent et al., 2017). In this paradigm, the reward is not expected at a specific point in time, so other mechanisms may be recruited by the LHb. A potential candidate for this process is the rebound activity of the LHb following inhibition by reward (Wang et al., 2017). However, alternating a target stimulus with reward delivery in an explicitly unpaired fashion also induces conditioned inhibition. There is ex-vivo evidence that such conditions promote meaningful activity in LHb neurons (Choi et al., 2020). In such case, learning could not be clearly linked neither to phasic decreases in dopamine in response to unexpected reward omission nor to postreward rebound activity of the LHb; therefore, other mechanisms involving the LHb may take place.

Converging lines of evidence suggest that LHb participates in the induction and expression of inhibitory learning under different conditions involving reward. This supports the intriguing idea that this structure is composed of several parallel circuits operating in different functionally equivalent situations (Stephenson-Jones et al., 2020). Unfortunately, conditioned inhibition, the accumulated outcome of inhibitory learning, is an elusive phenomenon which often requires special tests to be validated. Moreover, some authors have claimed that each test requires up to several control conditions to be deemed conclusive (Papini and Bitterman, 1993). This complicates the matter further, as it multiplies the number of subjects that are needed to evaluate the role of the LHb in this phenomenon; aside from comparing the role of its different subcircuits. However, this topic is still worth investigating, as it seems to participate in many situations involving negative predictive relationships between events. Perhaps, one situation in which such processes take place is the adaptation to environments that require behavior flexibility. If one takes “negative reward prediction errors” in a broader sense, many tasks requiring shifts in behavior following subtle cues could be considered in this category. Accordingly, there is an increasing amount of evidence that LHb plays a role in updating behavior on those tasks (Baker et al., 2017). Conditioned inhibition could be conceived as an accumulated outcome of the mechanisms involved in shifting behavior in dynamic conditions by error corrections. Models of associative learning would be useful to frame and test this and further hypothetical propositions.

## Footnotes

1 Note that the minimal response to the target stimulus observed after extinction procedures is quite labile and responding is often susceptible to bounce back after a slight contextual change (Bouton et al., 2021), this is not accounted by the Rescorla–Wagner model.

2 This paradigm is more broadly known as Pavlovian conditioned inhibition; however, “conditioned inhibition” is also used to designate the process that the target stimulus undergoes (here termed “inhibitory learning”), as well as the empiric demonstration that such a process has taken place (Savastano et al. 1999); therefore, here we use the term “feature-negative discrimination” for the procedure and “inhibitory learning” for the process to avoid ambiguity.

## Synthesis

Reviewing Editor: Nathalie Ginovart, University of Geneva Decisions are customarily a result of the Reviewing Editor and the peer reviewers coming together and discussing their recommendations until a consensus is reached. When revisions are invited, a fact-based synthesis statement explaining their decision and outlining what is needed to prepare a revision will be listed below. The following reviewer(s) agreed to reveal their identity: Phillip Baker, Manuel Mameli.

Upon discussion, the two reviewers agreed that the manuscript is timely and provides important insights into the role of the lateral habenula in inhibitory learning and, as such, support the publication. However, both reviewers also thought that revisions could significantly improve the manuscript. Most importantly, there was a consensus that the paper is way too long and that several sections need to be shortened and the information streamlined. Along this line, the authors should narrow the paper to focus more precisely on the role of the lateral habenula in learning process resulting from reward omissions.

Please find the specific comments and queries raised by the reviewers below:

<b>Reviewer 1:</b>

Ultimately, this review contains important insights into the role of LHb in Pavlovian inhibitory learning. In particular, it uses insight from both behavioral and physiological research stretching from classic models of reinforcement learning to recent calcium imaging in behaving animals. However, in its current form, the scope of the paper far exceeds both the necessary background required to establish a role for the LHb in inhibitory learning, and the immediate context in which the proposed role is applicable. It has the feel of a dissertation with the experimental chapters (in which the proposed careful examinations of LHb function during inhibitory learning and cue contrast experiments) removed. Implications such as inclusion of the LHb in artificial neural network models and a role in complex decision-making under instrumental learning conditions are certainly important but go beyond the proposed scope of the seeming goal of the review. In many ways, the forest gets lost for the trees in this manuscript and the main point is often lost. For example, several pages are spent discussing the Bush/Mosteller model of reinforcement only to dismiss it in favor of Rescorla/Wagner. Perhaps just not include it in the first place. In addition, while to connection between the Rescorla/Wagner model and observed results in inhibitory learning are variously included, a more succinct treatment of the specific variables that do and do not account for observed results in the LHb might clarify the point of including both in the manuscript with the overall goal of clarifying how the LHb contributes to reinforcement learning. There is also a major concern regarding various transitions between Pavlovian and instrumental behavior generally throughout the manuscript. The specifics of the proposed role of the LHb is stated to be in terms of Pavlovian behavior but at times, contrasting results are given in freely behaving animals in instrumental conditions. It at times appears that the authors suggest the role they are proposing applies to both forms of behavior while at other times, they note that in these conditions, the LHb appears to have a more complex role in behavioral selection than simply signaling reward omission (for example in the role of DA beyond reward signaling section). The paper should be narrowed to focus more specifically on Pavlovian behavior with a shorter section on understanding how it may apply to instrumental behaviors. To largely summarize, I found the overall premise of this review to be an interesting contribution but it requires significant revision to narrow the focus onto the proposed role in Pavlovian inhibitory learning through omission processing rather then attempting to combine the entire field of LHb behavior without properly accounting for results in freely behaving animals such as in the recent paper by Lecca et al. and earlier work. The authors continually narrow complicated results such as bidirectional signaling of reward or punishment, reinforcement and omission, and Pavlovian and instrumental behaviors to only consider results that reinforce the proposed role.

Specific comments below:

One crucial caveat to much of the assumptions given in the present paper, is the lack of accounting for neural activity in freely behaving animals. Prior papers have associated velocity correlations and behavior locking to LHb neural activity (Lecca et al. 2020, Baker et al. 2015, Sharp et al. 2006). How does the largely positive correlations of neural activity in LHb to movement associated behaviors square with the proposed role in inhibiting behavior?

In general section 4 contains a lot of conjecture about inputs with out adding to what has already been largely proposed in prior work by Stephenson-Jones and Hikosaka’s group.

In some ways, the proposed mechanism of LHb involvement in conditioned recognition is an instance of what has already been proposed for the LHb in context recognition going back to early papers from the 60’s. Early lesion work in aversive escape in unstable conditions demonstrates how changes the valence of cues fails to be signaled with a habenular complex. A short integration of those findings could be informative to the current proposal.

Paragraph in the 400’s about prelimbic fails to recognize the strong association of the PL with switching behaviors in appetitive environments as well which could account for the observed effects. Ragozzino summarized some of these findings in the PNAS paper. So did Moghaddam in several electrophysiology papers. In fact, Laubach’s, Floresco’s work and more have added important insights into the role of mPFC in attention and switching behaviors across diverse types of cues and contexts. The two papers cited to discount this larger role seem insufficient to discount work done over the past 40 years on the role of the mPFC in complex decision-making. Certainly, inhibitory control is an important aspect but it seems to be congruent rather than opposed to the role in hierarchical control at least in rodents. On a slightly less important not, the equivalency of the PL cortex in rodents with the DLPFC in humans is certainly controversial (See Wise, or Baxter, or Laubach for example) and I’m not sure what it adds to your paper. This relates in some ways to the differences between rodent and primate PFC generally and how the specific roles end up playing out with primate regions in some cases having no equivalent in rodents and roles sometimes being confused between species. I would suggest instead simply talking about the PL in this case at it has homology with human regions of agranular cortex and this likely is still applicable to human function without raising such a controversial issue.

The sentence on line 41-43 is vague.

Line 44 is also vague. What is meant by “an interphase?” Particularly in reference to the Vento and Jhou preview article. In that article they are talking about the bidirectional coding of valence.

Line 57 needs to be more specific. How does this paper support your hypothesis? These results transfer either to or away from the negative occasion.

If the hypothesis is that is in involved in RPEs then this has also be heavily reviewed. I think you need to contrast your proposal with the work on RPE signaling through the LHb here (line 67).

Perhaps an important additional consideration in the nature of changes in reward signaling of DA neurons when the mPFC. The work by Jo, Lee, and Mizumori (2013) could help clarify your proposed role of the mPFC in updating expectations as animals learn.

<b>Reviewer 2:</b>

This comprehensive review article mainly wants to focus on the contribution of the epithalamic lateral habenula to learning processes stemming from reward omission.

In order to tackle this issue, the authors extensively describe and report the experimental evidence that underlie reward encoding as well as reward prediction error computation, to then integrate how the lateral habenula plays a role within this framework.

This work is timely and stands out from other review articles on the topics that have been recently published. This increases the originality of this work. The writing is clear. However, I find the entire manuscript too lengthy and some of the arguments are circular. Streamlining some of the concepts and providing an overall take home message in each section would be helpful for the reader. The portion on mental health is important, yet it is not clear how the authors want to bridge the reward omission control by lateral habenula and disease states. In several instances, the discussion about the pathological side is too detached from the LHb itself and becomes too speculative. I would either build a comprehensive link between reward omission encoding in the LHb and pathological states, or rather eliminate the section.

Overall, the article is interesting and I believe it would be of reference for scientists in the field and for experts in lateral habenula function. Before publication, however, I do recommend that the authors address the following points:

- Reward omission encoding in the LHb field is dominated by the nomenclature of reward prediction error (RPE). The authors could relate their Equation 2 to current definitions of RPE (i.e., difference between expected an actual outcome).

- Line 281: Shabel reference is incorrectly used. The appropriate one is Stamatakis et al., 2016.

- The experiments performed by Donaire et al., 2019 and Zapata et al., 2017 (line 131-132) suggest a role for the LHb in extinction. In the experiments performed by Laurent et al., 2017 (line 328) there is also an extinction component when lever pressing is no longer followed by reward delivery. The authors could explain better the difference between conditioned inhibition and extinction, and how the results from Laurent et al. relate to those of Donaire and Zapata.

- For the purpose of clarity, the authors could better explain the behavioral paradigm employed by Choi et al., 2020 (line 389).

- The authors should consider eliminating the passage from lines 445-494, since a direct link to LHb physiology is somewhat lacking.

- The authors must include to their reference list Stopper and Floresco (2014), and discuss their implications in reward omission encoding.

- The reference of Cui et al., 2018 (line 552) is improperly used. The authors must either rephrase the sentence or eliminate the passage.
